# High-throughput screening of metal-porphyrin-like graphenes for selective capture of carbon dioxide

**DOI:** 10.1038/srep21788

**Published:** 2016-02-23

**Authors:** Hyeonhu Bae, Minwoo Park, Byungryul Jang, Yura Kang, Jinwoo Park, Hosik Lee, Haegeun Chung, ChiHye Chung, Suklyun Hong, Yongkyung Kwon, Boris I. Yakobson, Hoonkyung Lee

**Affiliations:** 1 School of Physics, Konkuk University, Seoul 143-701, Korea; 2 Department of Physics and Graphene Research Institute, Sejong University, Seoul 143-747, Korea; 3 Department of Chemistry, Ulsan National Institute of Science and Technology, Ulsan 689-798, Korea; 4 Department of Environmental Engineering, Konkuk University, Seoul 143-701, Korea; 5 Department of Biological Sciences, Konkuk University, Seoul 143-701, Korea; 6 Department of Materials Science and Nanoengineering, Rice University, Houston, Texas 77005, United State

## Abstract

Nanostructured materials, such as zeolites and metal-organic frameworks, have been considered to capture CO_2_. However, their application has been limited largely because they exhibit poor selectivity for flue gases and low capture capacity under low pressures. We perform a high-throughput screening for selective CO_2_ capture from flue gases by using first principles thermodynamics. We find that elements with empty *d* orbitals selectively attract CO_2_ from gaseous mixtures under low CO_2_ pressures (~10^**−**3^ bar) at 300 K and release it at ~450 K. CO_2_ binding to elements involves hybridization of the metal *d* orbitals with the CO_2_ π orbitals and CO_2_-transition metal complexes were observed in experiments. This result allows us to perform high-throughput screening to discover novel promising CO_2_ capture materials with empty *d* orbitals (e.g., Sc– or V–porphyrin-like graphene) and predict their capture performance under various conditions. Moreover, these findings provide physical insights into selective CO_2_ capture and open a new path to explore CO_2_ capture materials.

Carbon dioxide gas is a greenhouse gas that is a primary cause of global warming, which is known to cause severe climate change^1^. In recent years, the temperature of the earth has increased because of significant increase in CO_2_ emission. The emission of this gas is expected to continuously increase as the demand for fossil fuels increases, and thus the development of technologies for CO_2_ capture is essential for addressing climate change^1^. The technology involving the capture of CO_2_ gas from the flue gas is currently not sufficiently developed, particularly in the backdrop of the urgent need to reduce CO_2_ emission.

Nanostructured materials, such as graphene, zeolites, and metal-organic frameworks, have been considered to capture CO_2_. These materials are potentially useful because of their high capacity, fast CO_2_ adsorption kinetics, and effective regeneration^2^,^3^,^4^,^5^,^6^,^7^,^8^,^9^,^10^,^11^. However, their application has been limited largely because they exhibit poor selectivity for flue gases and low capture capacity under low pressures (~10^−3^ bar)^11^,^12^,^13^,^14^, thereby limiting CO_2_ capture from flue gases in power plants^14^. Thus, there is an increasing demand to search for novel CO_2_ capture materials^15^,^16^,^17^.

Recently, Fe–porphyrin-like fragments (FeN_4_) to carbon nanotubes^18^ and Co-porphyrin-like fragments (CoN_4_) to nanostructures^19^ were synthesized using the chemical vapour deposition and the pyrolysis methods, respectively, where Fe or Co is located at the center of four nitrogen atoms similar to metal-porphyrin structure^20^,^21^. We herein refer to this MN_4_ structure as an M–porphyrin-like structure. Fused transition metal (TM)-porphyrin-like nanoclusters have been synthesized experimentally^22^,^23^,^24^,^25^,^26^. Furthermore, the porphyrin-like structure is analogous to the local structure of Fe in hemoglobin^27^ or myoglobin^28^, which deliver O_2_ to the organs in the body. The concentration of nitrogen in carbon nanotubes and graphene has been found to reach ~8%^29^ and ~10%^30^, respectively. Thus, we expect that TM–porphyrin-like nanostructures can be synthesized experimentally. In this article, we perform first-principles thermodynamics based high-throughput screening for suitable M elements as selective CO_2_ attractors using M–porphyrin-like graphene.

## Results

To measure the CO_2_ capture capabilities of nanomaterials from a mixed gas, we constructed a thermodynamic model of CO_2_ adsorption on an adsorbent using the grand-canonical partition function^31^. We assumed a surface containing the number of identical, independent, and distinguishable adsorption sites (N_s_) with no mixed adsorption of different molecules per adsorption site, wherein the number of adsorbed *i*-type gas molecules on the surface is 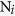. If the adsorbed molecules and gases are in equilibrium, the grand partition function of the system can be written as


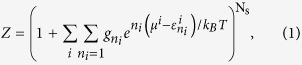


where superscript *i* indicates the type of gas, 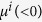 denotes the chemical potential of the *i*-type gas, and 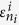
(<0) and 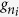 denote the average adsorption energy and degeneracy of configuration (for a given adsorption number *n*_*i*_) of the *i*-type gas molecules, respectively. When the thermally average number of *i*-type CO_2_ is calculated from 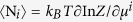, the occupation function (i.e., coverage) of CO_2_ for an adsorption site can be written as


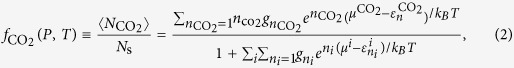


Therefore, the thermodynamic CO_2_ capture capacity of nanomaterials from a mixed gas can be computed using


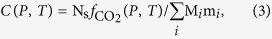


where M_*i*_ and m_*i*_ denote the atomic mass and number of elements comprising the adsorbent, respectively.

**Figure 1:**
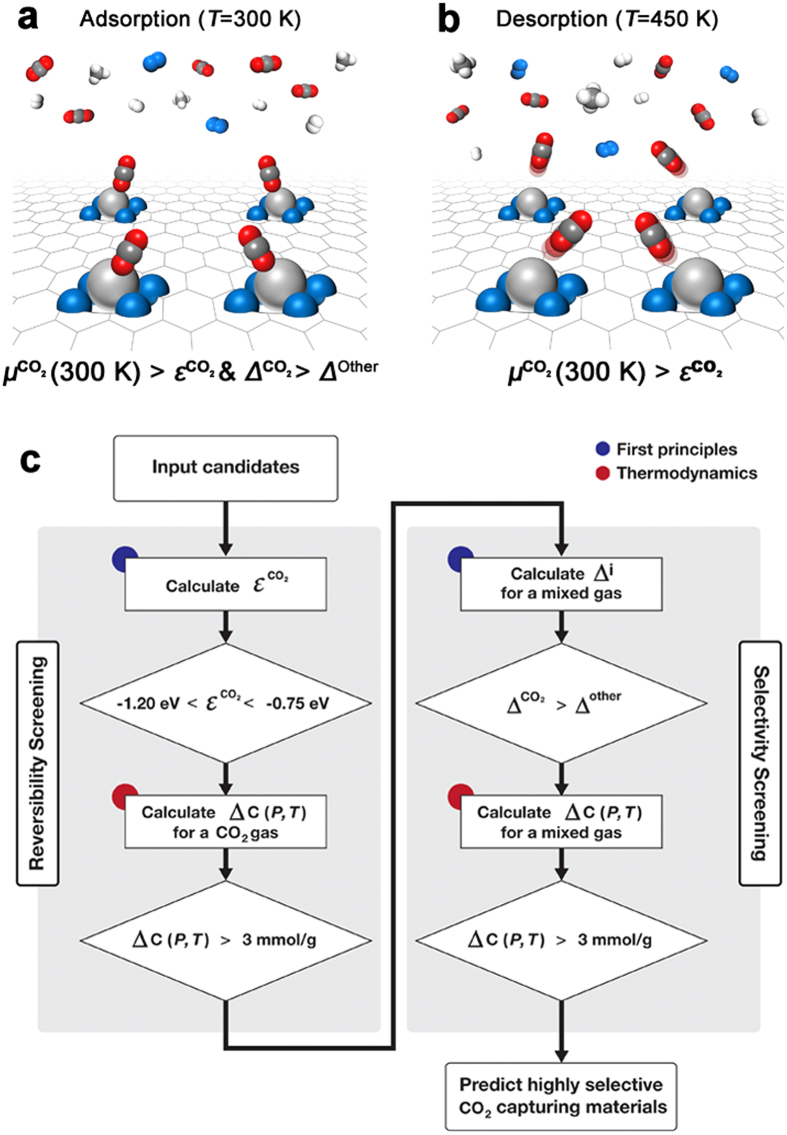
Thermodynamics of reversible/selective adsorption of CO_2_ and flow chart for predicting selective CO_2_ capture materials: (**a**) Selective CO_2_ adsorption occurs through competitive adsorption between CO_2_ and other molecules if 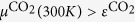 and 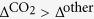. (**b**) CO_2_ molecules adsorbed on the metal sites are released if 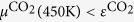. (**c**) Flow chart for predicting reversible and selective CO_2_ capture materials based on first principles thermodynamics: this consists of reversibility screening for pure CO_2_ gas and selectivity screening for a mixed gas.

The occupation function of CO_2_ would have a positive value, i.e., 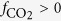, if 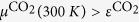 and 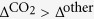 at the adsorption (capture) conditions as shown in Fig. 1a, wherein 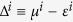 is set and the superscript ‘other’ denotes molecules other than CO_2_. In this case, selective CO_2_ adsorption occurs through competitive adsorption between CO_2_ and other molecules; this is attributed to the fact that the Gibbs factor for CO_2_ adsorption is much greater than unity and the Gibbs factors of other molecules, i.e., 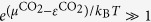 and 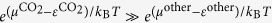. However, the occupation function would be zero, i.e., 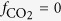, if 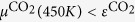, at the desorption (release) conditions (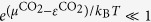) as shown in Fig. 1b, indicating that CO_2_ adsorbed on the metal sites is released. Under a CO_2_ pressure of ~10^**−**3^ bar, the ideal conditions for adsorption and desorption are assumed to be 300 and 450 K, respectively, where 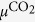 is approximately −0.75 and −1.20 eV, respectively, at ambient conditions. Thus, the key thermodynamic conditions for reversible and selective CO_2_ capture from a mixed gas are as follows: (i) 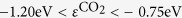 and (ii) 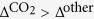.

From this we construct a computational approach to efficiently predict selective CO_2_ capture materials based on first principles thermodynamics shown in Fig. 1(c). The thermodynamic conditions and capacity requirements^11^ for screening are as follows: 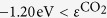 < 0.75 eV and 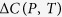 > 3 mmol g^−1^ for CO_2_ gas, and 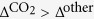 and 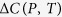 > 3 mmol g^−1^ for a mixed gas. 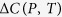 denotes the difference between 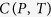 at 300 K and 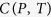 at 450 K under a pressure of 10^**−**3^ bar, which indicates the CO_2_ working capacity. These requirements may need to be revised depending on the operational environments.

**Figure 2:**
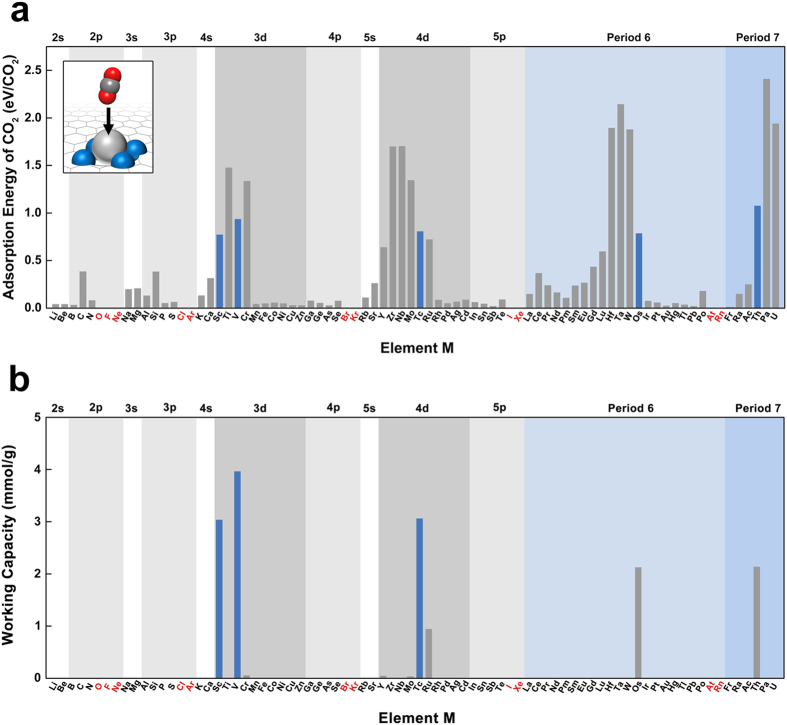
Reversibility screening of many candidates: (**a**) Calculated adsorption energies of CO_2_ molecules on M–porphyrin-like graphene and a variety of nanostructures. Inset shows the schematic of CO_2_ binding to the M site and colored-marked elements indicate data not available. (**b**) Calculated CO_2_ capture capacity, *C*(*P,T*), on M–porphyrin-like graphene for CO_2_ gas at 300 K under a CO_2_ pressure of 10^−3^ bar. Colored-marked bars indicate candidates which meet the requirements.

We performed calculations on the adsorption energy of CO_2_ molecules on the M sites of M–porphyrin-like graphene (Fig. 2a). Elements of atomic numbers up to 92 for the M site were considered, and the others were ruled out because of their heavy weight. Sc–, V–, Tc–, Os–, and Th–porphyrin-like graphenes out of many candidates met the reversibility requirements, viz. −1.2 to −0.8 eV (Fig. 2a), where a CO_2_ molecule adsorbs on a TM atom with the distance of ~2.5 Å between the TM atom and the CO_2_ molecule. Therefore they were considered for the next step. We also performed CO_2_ adsorption calculations on carbon allotropes such as carbon nanotubes, graphene, and C_60_. The adsorption energy of the CO_2_ molecule is ca. −0.05 eV, and the distance between their surface and the molecules is ~3.5 Å. In this case, since the adsorption energy of CO_2_ molecules is much smaller than the required adsorption energy, pristine carbon nanostructures may not be suitable for use as CO_2_ capture media under low pressure at room temperature. Notably, our approach significantly reduces the computational load because it is not necessary to calculate 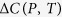 for all the candidates in CO_2_ gas or a mixed gas.

To predict the capture capabilities of the candidates, the CO_2_ working capacities, 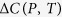, of the structures were computed using Eq. (3) (Fig. 2b). The experimental values of the chemical potentials of CO_2_ gas and calculated adsorption energies (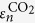) were used in these calculations. Since the working capacities of Sc–, V–, and Tc–porphyrin-like graphenes satisfied the capacity requirement (>3 mmol g^**−**1^), they were considered for the next selectivity screening step.

**Figure 3:**
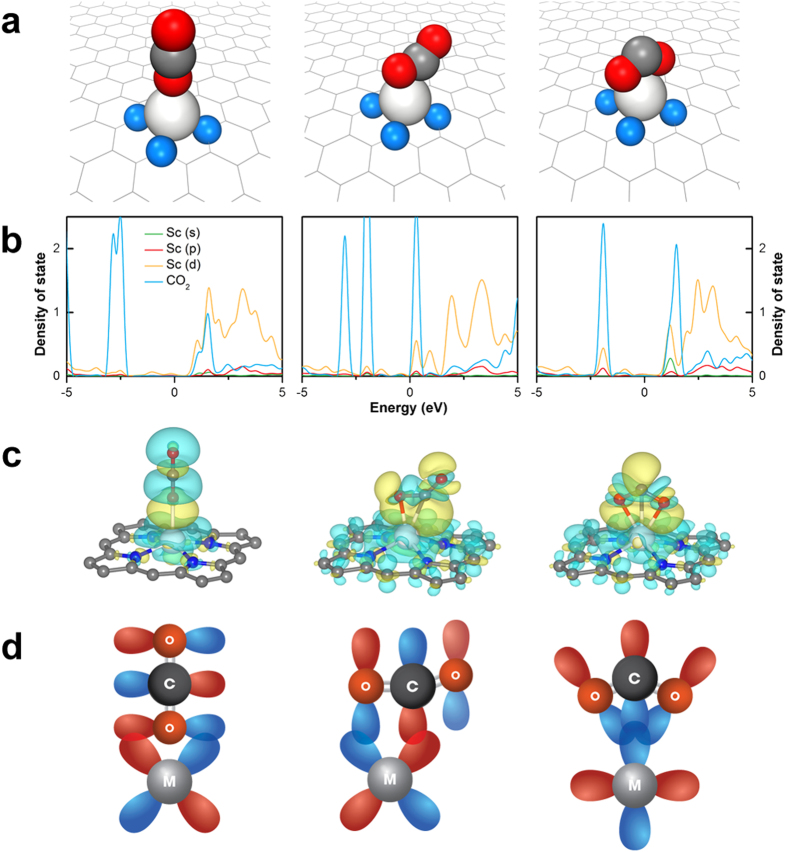
Origin of distinct geometries of CO_2_ adsorption: (**a**) Atomic structures showing CO_2_ molecule adsorbed onto Sc–4N graphene for the various CO_2_ adsorption geometries designated as η^1^-CO_2_, η^2^-CO_2_, and η^3^-CO_2_, respectively. (**b**) The density of states for η^1^, η^2^, and η^3^ geometries, respectively. (**c**) The difference in the total charge density 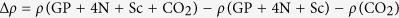 for η^1^, η^2^, and η^3^ geometries, respectively. Yellow and green indicates the charge accumulation and depletion. (**d**) The schematic of the hybridization of the Sc 3*d* orbitals with the CO_2_ *p*_z_ orbitals for η^1^, η^2^, and η^3^ geometries, respectively. Red and blue colors of the orbitals indicate the different phases, respectively.

We observed three different geometries for the adsorbed CO_2_ molecules on the TM atoms, which were designated as η^1^-CO_2_, η^2^-CO_2_, and η^3^-CO_2_, corresponding to the coordination numbers of the TM atom, i.e., 1, 2, and 3, respectively (Fig. 3a). The adsorption energies of the CO_2_ molecules were calculated to be −0.54, −0.79, and −0.78 eV per CO_2_ for the Sc-η^1^-CO_2_, Sc-η^2^-CO_2_, and Sc-η^3^-CO_2_ geometries, respectively. The preferred CO_2_ geometry depends on the metal type. The distance between the CO_2_ molecule and TM atoms is 2.2–2.5 Å, which is much smaller than the equilibrium van der Waals distance (~3.4 Å), and the bond lengths of CO_2_ are elongated by ~5%. Thus, the bonding between the TM atoms and CO_2_ molecules must be chemical in nature.

To understand the enhanced interaction between early *d* orbital–containing elements and CO_2_ molecules, we focused on a binding mechanism that appears between TM atoms and olefin molecules and is well known in organometallic chemistry^32^. The Dewar–Chatt–Duncanson model explains the type of chemical bonding between a π-orbital acid alkene and *d*-orbital metal atom by electron donation (i.e., hybridization of the empty *d* states with filled π states) and back-donation (i.e., hybridization of the filled *d* states with empty π states)^32^. The interaction between the TM *d* orbitals and the olefin π orbitals is called the “Dewar interaction”. Therefore, empty *d*-orbital metals are expected to attract CO_2_ molecules. The Dewar interaction is based on chemical bonding between the TM and CO_2_ and can enhance the strength of the M–CO_2_ bond beyond that of the van der Waals interaction. It is noteworthy that Ca^2+^ also has empty 3*d* orbitals near the Fermi level that could participate in the Dewar interaction.

Next, we investigated whether the enhanced adsorption observed with early TM atoms is caused by the Dewar interaction. We observed the hybridization of the Sc 3*d* states with the CO_2_ states at around −2.5, −2.0, and −2.0 eV for the η^1^-CO_2_, η^2^-CO_2_, and η^3^-CO_2_ geometries, respectively (Fig. 3b). The difference in charge density between the Sc atom and CO_2_ molecule (Fig. 3c) indicates chemical bonding between CO_2_ and the metal atoms. From this, we concluded that the enhanced binding of CO_2_ to the metal atom originates from the Dewar interaction. The distinct adsorption geometries of CO_2_ can be explained by the different hybridization states of the TM *d* orbitals with the CO_2_ π orbitals (Fig. 3d).

**Figure 4:**
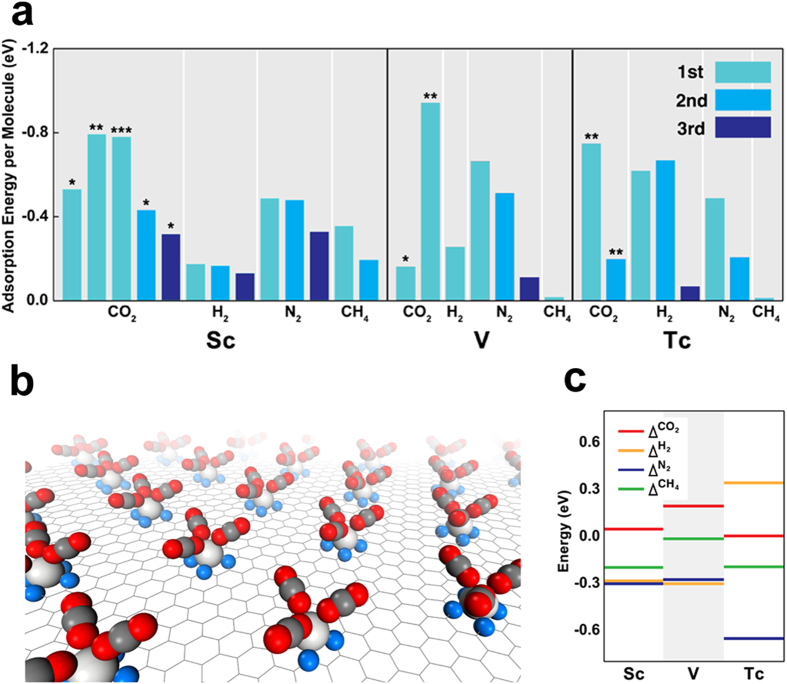
Selectivity screening by selective CO_2_ capture condition: (**a**) Calculated (average) adsorption energies of molecules for the different types of molecules with different numbers of the molecules as TM atoms (TM = Sc, V, Tc). *, **, and *** indicate the geometric configurations of η^1^, η^2^, and η^3^, respectively. (**b**) Optimized geometry of three CO_2_ molecules adsorbed onto a Sc atom of Sc–porphyrin-like graphene with the η^1^ configuration. (**c**) The difference (
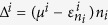
) between the chemical potential of a gas and adsorption energy of the gas molecule on TM–porphyrin-like graphene with respect to the type of gas. The largest values of 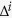 were chosen regardless of 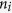.

**Figure 5:**
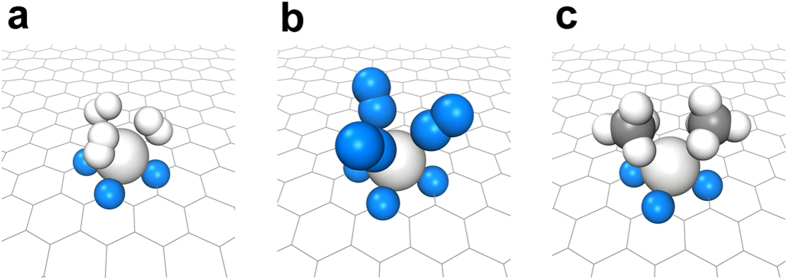
Adsorption of various molecules on Sc-porphyrin-like graphene: **(a**) Up to three H_2_ molecules adsorb on a Sc atom. (**b**) Up to three N_2_ molecules adsorb on a Sc atom. (**c**) Up to two CH_4_ molecules adsorb on a Sc atom.

To examine the selectivity of CO_2_ adsorption on Sc, V, and Tc sites in the presence of a mixed gas, we also carried out calculations on the adsorption of multiple CO_2_ molecules or ambient gas molecules such as N_2_, CH_4_, and H_2_ onto the metal atoms. Several CO_2_, H_2_, N_2_, and CH_4_ molecules bound to Sc, V, and Tc atoms (Figs 4a,b and 5). The difference between the chemical potential at 300 K and 10^**−**3^ bar and the adsorption energy of CO_2_ (or other gas molecules) was calculated (Fig. 4c) using experimental values of the chemical potentials of CO_2_, H_2_, N_2_, and CH_4_ gases. The chemical potentials of gases were obtained by fitting the experimental values to the following expression 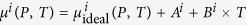 where upper subscript *i* indicates the type of gases, 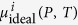 denotes the chemical potential of an ideal monatomic *i*-type gas for a given the pressure *P* and the temperature *T*, and 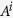 and 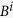 are fitted coefficients of *i*-type gas. The fitted coefficients are presented in Table 1. Since Sc and V, but not Tc, were found to satisfy the conditions for selective CO_2_ adsorption (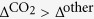), they were considered for the next screening step.

We also considered the zero-point vibrational energy of the gas molecules adsorbed onto the TM atoms. This energy was calculated to be in the order of a few meV regardless of the metal. Since the zero-point vibrational energy is negligible compared to the (static) adsorption energy (Fig. 4a), we ignored the influence of the zero-point vibrational energy on adsorption in all cases except for H_2_. Since the zero-point energy of the H_2_ molecules adsorbed on TM atoms was not negligible (25% of the calculated values), we corrected the H_2_ adsorption energies to determine the true adsorption energy.

The statistical model obtained here can correctly describe the adsorption of CO_2_ onto TM–porphyrin-like graphene in the presence of a mixed gas because the mixed adsorption of different molecules onto a TM atom is not energetically favorable. For instance, the adsorption energy at which both a CO_2_ and N_2_ molecule adsorb onto a Sc atom was calculated to be −0.9 eV, which is much higher than that (−1.3 eV) at which single CO_2_ or N_2_ molecules adsorb on different sites.

**Figure 6:**
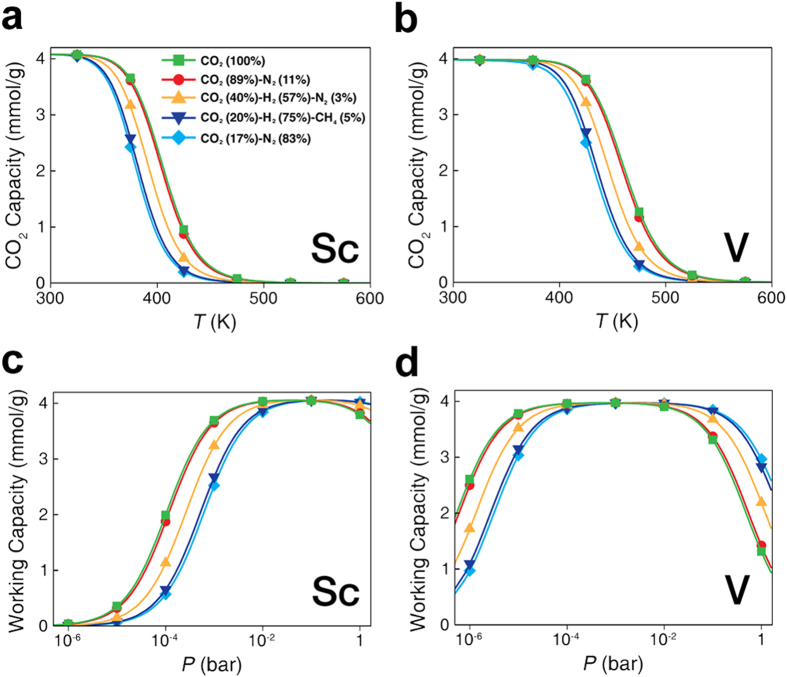
Selectivity screening by CO_2_ working capacity: Calculated capacities, 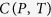, of CO_2_ of the TM–porphyrin-like graphenes as a function of temperature under total pressure, 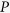, of 10^−3^ bar using Eq. (3): (**a**) Sc–porphyrin-like graphene and (**b**) V–porphyrin-like graphene. The following different compositions of gases were considered: Pure CO_2_ (100%), CO_2_ (89%)-N_2_ (11%), CO_2_ (40%)-H_2_ (57%)-N_2_ (3%), CO_2_ (20%)-H_2_ (75%)-CH_4_ (5%), and CO_2_ (17%)-N_2_ (83%). The partial pressure of gases is given by 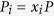, where 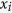 is the composition of the gas. Calculated working capacities of CO_2_ in the TM–porphyrin-like graphene as a function of the total pressure, 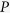, of the gases from 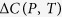, the difference between 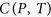 at 300 K and 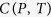 at 450 K: (**c**) Sc–porphyrin-like graphene and (**d**) V–porphyrin-like graphene.

The CO_2_ capture capacities, *C*(*P*, *T*), from mixed gases with different compositions were calculated for Sc– and V–porphyrin-like graphenes (Fig. 6a,b). The ratios of the mixed gases were based on experimental measurements^4^,^33^ from pre-combustion, post-combustion, and oxyfuel-combustion CO_2_ capture. These results show high CO_2_ selectivity of Sc– and V–porphyrin-like graphene in mixed gases, which is consistent with the prediction of the selectivity requirement of 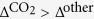. The CO_2_ working capacities, 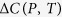, of Sc– and V–porphyrin-like graphenes can reach ~4 mmol g^**−**1^ (Fig. 6c,d), which meets the capacity requirement of 3 mmol g^**−**1^ in a mixed gas. Therefore, Sc– and V–porphyrin-like graphene were found to be suitable for highly selective CO_2_ capture from flue gases at ambient conditions. Furthermore, the CO_2_ pressure range covers the pressure (~0.4 × 10^−3^ bar) of CO_2_ in the atmosphere because the concentration of CO_2_ in the atmosphere is ~400 ppm.

## Discussion

We performed first-principles total energy calculations regarding CO_2_ adsorption onto metal–porphyrin-like structures to explore the feasibility of achieving room-temperature CO_2_ capture under low pressures. We found that transition metal–porphyrin-like structures adsorb CO_2_ molecules with the desirable binding energy range and the practical (usable) capacity under ambient conditions can reach ~3 mmol/g. Equilibrium thermodynamics studies showed that Sc– or V–porphyrin-like graphene structures were found to be suitable for use as room-temperature CO_2_ capture media. These results indicate that nanostructures containing empty *d* orbitals may be applied for selective adsorption of CO_2_ from flue gases. We believe our results provide a new approach to achieving CO_2_ capture at room temperature.

We address the evidence of CO_2_ binding to TM atoms for CO_2_ capture. TM-η^1^-CO_2_ or TM-η^2^-CO_2_ complexes were observed in experiments^34^,^35^. The capture of CO_2_ involved in the first step of carbon capture/storage (CCS) technology requires high energy consumption^36^,^37^. Thus, the development of media such as TM–porphyrin-like graphene nanostructures, which can selectively adsorb CO_2_ at room temperature under low CO_2_ partial pressure, is expected to lower the cost of CO_2_ adsorption and make CCS more viable.

## Methods

We performed first-principles calculations based on the density functional theory (DFT)^38^ as implemented in the Vienna Ab-initio Simulation Package (VASP) with the projector augmented wave (PAW) method^39^. The generalized gradient approximation (GGA) in the Perdew–Burke–Ernzerhof scheme^40^ was used for the exchange correlation energy functional, and the kinetic energy cutoff was taken to be 800 eV. For calculations of gas molecule adsorption, our model for the graphene-based system comprised a 3 × 3 hexagonal supercell, and the composition of the supercell was C_12_N_4_M_1_. Geometrical optimization of the graphene-based system was carried out until the Hellmann–Feynman force acting on each atom was less than 0.01 eV/Å. The first Brillouin zone integration was performed using the Monkhorst–Pack scheme^41^. 4 × 4 k-point sampling was used for the 3 × 3 graphene supercells. The chemical potential of gases, 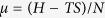, where *H*, *S*, and *N* denote the enthalpy, the entropy, and the number of particles was calculated from the data of the enthalpy (*H*) and entropy (*S*) in the reference: http://webbook.nist.gov/chemistry/fluid/.

## Additional Information

**How to cite this article**: Bae, H. *et al*. High-throughput screening of metal-porphyrin-like graphenes for selective capture of carbon dioxide. *Sci. Rep*. **6**, 21788; doi: 10.1038/srep21788 (2016).

## Figures and Tables

**Table 1:** The fitted chemical potentials of gases.

Gas type	*A*^*i*^ (eV)	*B*^*i*^ (eV/K)	R^2^
CO_2_	0.04271	−0.6425	0.99882
H_2_	0.02784	−0.1585	0.99590
N_2_	0.03000	−0.4512	0.99948
CH_4_	0.04868	−0.4840	0.99823

R^2^ is the coefficient of determination (measure of goodness of fit).
