# Corrigendum: High-throughput screening of metal-porphyrin-like graphenes for selective capture of carbon dioxide

**DOI:** 10.1038/srep46902

**Published:** 2017-09-13

**Authors:** Hyeonhu Bae, Minwoo Park, Byungryul Jang, Yura Kang, Jinwoo Park, Hosik Lee, Haegeun Chung, ChiHye Chung, Suklyun Hong, Yongkyung Kwon, Boris I. Yakobson, Hoonkyung Lee

Scientific Reports 6: Article number: 21788; 10.1038/srep21788 published online: 02 23 2016; updated: 09 13 2017.

This Article contains an error in Table 1, where ‘*B*^*i*^ (eV/K)’ should read ‘*B*^*i*^ (meV/K)’.

